# Cardiovascular disease risk in adults with compromised bone health

**Published:** 2023-01

**DECEMBER 05, 2022** - Adolescent and young adult (AYA) cancer survivors are at risk of experiencing treatment-related effects later in life, including damage to the heart. New research has identified various sociodemographic and modifiable risk factors associated with these patients’ risk of developing cardiovascular disease (CVD). The findings are published by Wiley online in CANCER, a peer-reviewed journal of the American Cancer Society.

The study by investigators at Duke University and The University of Texas MD Anderson Cancer Center relied on 2009-2018 data from the National Health Interview Survey, which collects information on a broad range of health topics through personal interviews of US households. Responses from 4,766 AYA cancer survivors and 47,660 controls (without a history of cancer) were included.

The risk of CVD was significantly higher in survivors than controls by sex, race/ethnicity, income, education, smoking status, and physical activity. Also, household income <$50K/year disproportionately increased the odds of CVD in survivors compared with controls.

In the AYA survivor population, male sex, Black race, household income <$50K/year, and current or former smoking were all associated with higher odds of CVD. Performing any moderate to vigorous intensity physical activity was associated with lower CVD odds.

“These results highlight the importance of long-term surveillance of AYAs after cancer treatment to ensure that appropriate screenings are initiated to reduce the risk of CVD and to promote healthy behavioral changes, such as physical activity, which impact long-term CVD outcomes,” said lead author Amy Berkman, MD, of the Duke University School of Medicine.

*Full Citation: “Cardiovascular disease in AYA cancer survivors: impact of sociodemographic and modifiable risk factors.” Amy M. Berkman, Clark R. Andersen, Michael E. Roth, and Susan C. Gilchrist. CANCER; Published Online: December 5, 2022 (DOI: 10.1002/cncr.34505).*

*URL Upon Publication: http://doi.wiley.com/10.1002/cncr.34505*

*Copyright © 2021 The Cochrane Collaboration. Published by John Wiley & Sons, Ltd., reproduced with permission.*

